# Interactive Exploration of Longitudinal Cancer Patient Histories Extracted From Clinical Text

**DOI:** 10.1200/CCI.19.00115

**Published:** 2020-05-08

**Authors:** Zhou Yuan, Sean Finan, Jeremy Warner, Guergana Savova, Harry Hochheiser

**Affiliations:** ^1^ University of Pittsburgh, Pittsburgh, PA; ^2^ Boston Children’s Hospital, Boston, MA; ^3^ Vanderbilt University, Nashville, TN

## Abstract

**PURPOSE:**

Retrospective cancer research requires identification of patients matching both categorical and temporal inclusion criteria, often on the basis of factors exclusively available in clinical notes. Although natural language processing approaches for inferring higher-level concepts have shown promise for bringing structure to clinical texts, interpreting results is often challenging, involving the need to move between abstracted representations and constituent text elements. Our goal was to build interactive visual tools to support the process of interpreting rich representations of histories of patients with cancer.

**METHODS:**

Qualitative inquiry into user tasks and goals, a structured data model, and an innovative natural language processing pipeline were used to guide design.

**RESULTS:**

The resulting information visualization tool provides cohort- and patient-level views with linked interactions between components.

**CONCLUSION:**

Interactive tools hold promise for facilitating the interpretation of patient summaries and identification of cohorts for retrospective research.

## INTRODUCTION

The complexities of cancer care create significant challenges for the extraction of information for retrospective research. As patients progress through diagnosis to treatment and subsequent monitoring, multiple encounters with varying specialists generate a rich set of clinical notes. For patients undergoing lengthy or multimodal (eg, a combination of surgery, chemotherapy, and radiotherapy) treatment, hundreds or thousands of notes can be generated along the “cancer journey.” Review of these notes can be a laborious interpretive challenge, often involving many hours of time for medical professionals who must read through collections of notes to prepare summarized abstractions in spreadsheets or databases. This process is also brittle, as reviews conducted for one study may miss items of potential interest to subsequent studies. Although ad hoc solutions such as the “oncologic history” have spontaneously developed as information collection devices, they are not necessarily universal, accurate, or complete.^1^

**CONTEXT**

**Key Objective**
How can interactive tools help researchers and clinicians understand longitudinal histories of patients with cancer extracted from notes via natural language processing?
**Knowledge Generated**
Web-based views at both the patient and cohort levels can provide display summary and detail information about complex cancer cases. Interaction techniques linking views at different granularities can enable navigation between summaries and details.
**Relevance**
Interactive tools for exploring summary representations of cases provide the possibility of easing interpretation of complex details as needed to inform care or to drive translational research.

The Cancer Deep Phenotype Extraction (DeepPhe) project is developing informatics solutions to overcome these inefficiencies. Unlike prior work applying natural language processing (NLP) techniques to individual cancer documents,^2-5^ DeepPhe combines details from multiple documents to form longitudinal summaries. Classic and state-of-the-art NLP techniques for extracting individual concepts are used alongside a rich information model^6^ and techniques for care episode classification,^7^ cross-document coresolution,^8^ and rule-based inference to summarize diagnoses, treatments, responses, and temporal relationships as needed to support retrospective research.^9^ We expect that DeepPhe will be used either by clinicians or researchers with appropriate permissions to read notes de-identified by honest brokers or through other appropriate means. DeepPhe v3 was released in March 2019 and is available on GitHub.^10^

The application of the NLP tools to notes collected over months or years can lead to hundreds of observations: one modestly sized test data set of 49 patients had an average of > 245 facts/patient (standard deviation [SD], 99.3), spread over an average of 30.6 notes (SD, 18.4). Information visualization tools have the potential to help users easily interpret these rich records. The DeepPhe multilevel information model can easily support the “overview first, zoom and filter, details on demand”^11^ approach that has proven successful in many previous visualization efforts. In the case of DeepPhe, “details on demand” suggests drilling down from summarized representations to inference rules and specific spans of text that provide the provenance for those higher-level summaries.

Our goal is to develop a multiscale visualization to help researchers interpret the complexities of relationships between cancers, tumors, treatments, responses, biomarkers, and other key attributes. We draw on a substantial body of prior work on visual cohort extraction tools, many of which have used temporal or flow metaphors to characterize temporal trends or transitions across patient populations.^12-15^ The DeepPhe-Viz tool will extend these efforts with facilities for addressing challenges associated with the ambiguities of interpreting natural language. Our design of this tool was motivated by insights from qualitative inquiries with potential users and informed by our multilevel information model.

## METHODS

### Qualitative Inquiry

We conducted unstructured qualitative interviews with clinical cancer researchers at the University of Pittsburgh and Magee-Women’s Research Institute. Participants were a convenience sample identified through professional contacts of the research team. Interviews were conducted one on one, in participants’ workspaces, and covered a variety of questions focusing on challenges in cancer retrospective research, including goals, information needs, representations, bottlenecks, and challenges. Although contextual inquiry^16^ observations of researchers’ work as they reviewed clinical notes would have been preferred, interviewers did not have institutional review board clearance to see the de-identified patient data used by the researchers. Instead, interviews focused on general descriptions of the work and related challenges, including discussions of database schemas and tools such as spreadsheets used to manage extracted information.

All interviews were audio-recorded. Interviews were conducted and analyzed by a coauthor with extensive experience in human-computer interaction research (H.H.), using an emergent coding approach^17^ to extract information needs, problems, design suggestions, and other relevant information. Comments were specifically reviewed to identify user challenges, classified through emergent code into those involving information availability, access, quality, and interpretation. Results from these analyses were used to develop user personae describing potential DeepPhe users, user stories involving specific tasks, competency questions detailing specific information requirements, and flow diagrams describing user processes. The University of Pittsburgh Human Research Protection Office classified these inquiries as exempt (PRO13120154).

### Information Model

Qualitative inquiry results were used to develop an information model capable of representing relevant items and attributes at multiple granularities, ranging from individual text mentions to patient summaries.^6^

#### Mentions.

Text spans in source documents covering concepts of interest, including tumors, body locations, treatments, stage indicators, biomarkers, and other key elements. Mentions have individual properties, such as negation, uncertainty, and historicity.

#### Compositions.

Aggregations of mentions pertaining to the same unique entity or event. Composition Relations formulate clinical attributes and interconnection.

#### Episodes.

Collection of documents in key event intervals, initially including work-up, diagnosis, medical decision making, treatment, and follow-up.

#### Patient Summary.

Descriptions of cancers, tumors, treatments, and genomics, abstracted across the entire span of the patient history.

### Natural Language Processing

Apache cTAKES^18^ pipelines were extended to extract individual mentions of cancer information, populating the mention level of the model. Mentions from each document were aggregated and simplified via coreference resolution to form the composition level. Machine-learning models trained on annotated data are used to assign documents to episodes. Composition-level mentions are processed by a series of summarization rules to generate the high-level phenotypes. Results are stored in a Neo4j graph database.^19^ The initial DeepPhe architecture is described in detail by Savova et al.^9^

### Visualization

Insights from qualitative inquiries informed the software requirement specification along with a corresponding series of low-fidelity prototypes for the interactive tools and visualizations. Subsequent iterative improvements of the functional software were informed by feedback from translational cancer researchers, cancer registrars not directly involved in the DeepPhe project, and oncologists, including coauthor J.W. Revisions focused on enhancing the multiscale visualization capabilities (linking high-level summaries to individual text mentions)^11^ and improving the interactive coordination between various views.^20^

The DeepPhe-Viz tool was developed as a web application, using the Node.JS web platform,^21^ to provide a middle-ware layer capable of retrieving data through the Neo4j bolt protocol.^22^ The visualization interface was implemented in HTML, CSS, Javascript, and the D3 visualization toolkit.^23^ The DeepPhe-Viz tool is available on GitHub.^24^

## RESULTS

### Participants

Five researchers participated in the qualitative inquiries. Four had medical degrees, including two postdoctoral trainees, one practicing oncologist, and one full-time researcher. The fifth was a cancer epidemiologist with a PhD. Participants focused on either breast (n = 1) or ovarian (n = 4) cancer. Interviews were approximately 1 hour long.

### Qualitative Inquiry and Visualization Requirements

User challenges identified during interviews involved difficulties with information availability, access, quality, and interpretation. Although some issues were specific to the types of cancer or the context of care, most were more broadly applicable (Table 1).

**TABLE 1.**
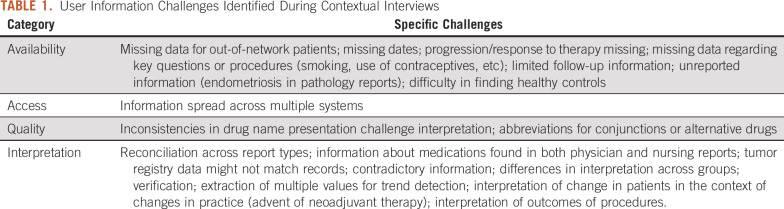
User Information Challenges Identified During Contextual Interviews

Together with informant descriptions of information needs and goals, these challenges informed the creation of user stories detailing specific tasks to be conducted for individual patients and/or at the cohort level. These user stories were broadly grouped into 14 requirement categories (Table 2).

**TABLE 2.**
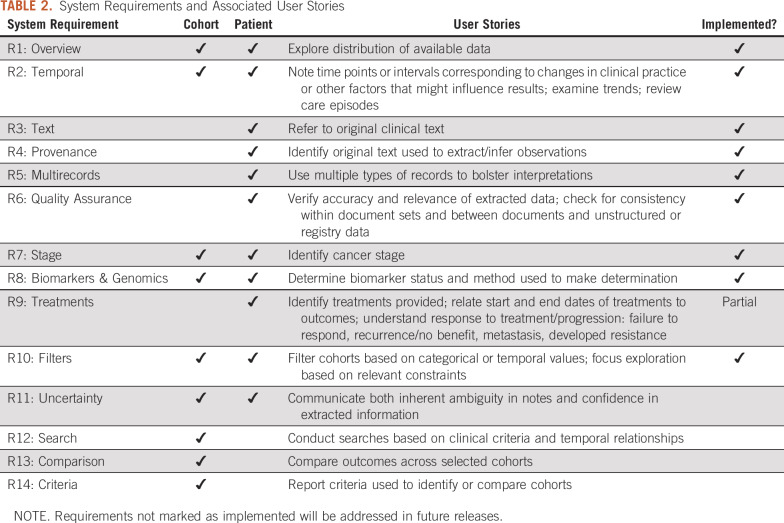
System Requirements and Associated User Stories

### Interactive Visualization Environment

As development of the DeepPhe NLP tools is an ongoing effort, prototype implementation of the visualization tools has been facilitated by the construction of synthetic details to complete fields that cannot yet be extracted by DeepPhe. The current prototype displays extracted results for cancer stage, diagnosis, treatments, tumor size, histologic type, tumor extent, cancer cell line, body site, and biomarkers. Synthesized results for date of birth and menopausal status are also displayed.

### Cohort View

**FIG 1.**
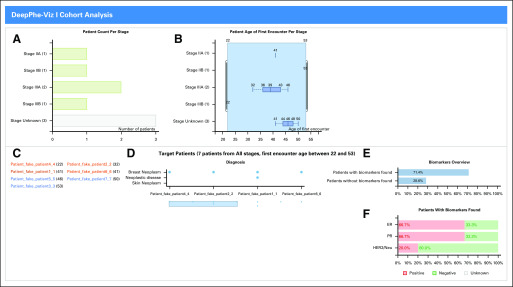
Cohort view: (A) Distributions of cancer stages, (B) distribution of ages, (C) scrollable list of patients, (D) distribution of diagnoses across patients, (E) distribution of biomarker counts, (F) status of three key breast cancer markers. As inference rules for summary staging are not included in DeepPhe (due to American Joint Committee on Cancer licensing requirements), a large portion of records are stage unknown.

The DeepPhe cohort viewer (Fig 1) provides multiple complementary views. Tumor stages are shown in two views: a simple histogram of diagnosis stages (Supporting R7; Fig 1A) and an age distribution box plot (Fig 1B). A list of patient names (Fig 1C) enables quick identification of specific patients, and a scrollable list of diagnoses associated with each patient (Fig 1D) facilitates comparison between patients. Biomarker views (Fig 1E and 1F) show which patients have identified biomarkers and the distribution of observations among the active members of the cohort. The stage histogram can also be used to focus on individuals with specific stages: clicking on one of the bars will update the histogram and all other components to show only those items matching the stated criteria (Filter, R10). The double-thumb slider on the patient age by stage view (Fig 1B) also acts as a filter (R10). Each of these views also provides an overview of the associated distributions (R1).

### Patient View

The DeepPhe-Viz patient view provides several panes at varying levels of granularity.

**FIG 2.**
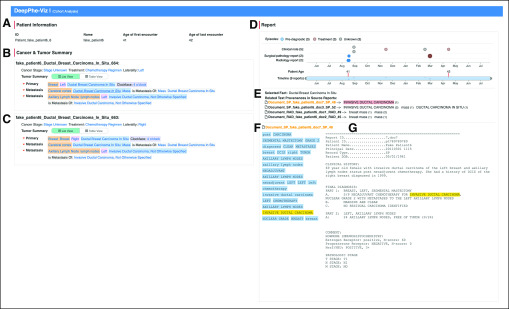
The patient visualization view, displaying manually fabricated synthetic data. (A) Patient demographics and cancer summary. (B, C) Cancer and Tumor summary for two different cancers. (D) Interactive timeline displaying documents by type and episode. (E) Inference provenance display. (F) Document-level mentions. (G) Clinical note. Selection of “Ductal Breast Carcinoma In Situ” in the cancer summary (B) leads to highlight of the first relevant note in the timeline. The provenance of this observation can be seen (E) in the display of relevant provenance rules, and (F) highlight of the selected term, (G) in the relevant document.

Under *patient details, cancer, and tumor*, overviews (R1) of patient demographics (Fig 2A) and cancer and tumor diagnoses (Fig 2B and 2C) are shown at the top left, providing a concise summary of patient details. Cancer details include summary cancer attributes, cancer stage (R7: Stage), treatments (R9: Treatments), cell line, and TNM values.^25^ The patient shown here has two independent cancer diagnoses, each providing overall stage summaries, treatments, and laterality, along with tumor details including specific diagnoses, biomarkers (R8: Biomarkers & Genomics), and other details shown as expandable lists of attributes colored to indicate classes of information. This approach provides a compact summary. A toggle at the top of the tumor summary pane supports switching to tabular views when desired. Tumor and cancer details can be selected to reveal individual text spans contributing to the summary element (R3: Text; R4: Provenance), thus providing an example of the use of the hierarchical model to go from summary to individual observation. Examination of these details can also be used to support R6: Quality Assurance.

The *clinical note timeline* supports R2: Temporal by displaying multiple types of notes (R5: Multirecords) on a timeline with one lane for each type of note (progress, radiology, and surgical pathology; Fig 2D). Notes are color-coded according to episode. A double-thumb scroll bar below the timeline allows zooming and panning across the extent, which spans from the interval between the first and last available documents. Episode labels above the timeline can be clicked to zoom the timeline to documents contained in the specified episode.

Below the timeline, the *explanation panel* (Fig 2E) supports R3: Text, R4: Provenance, and R6: QA by bridging the gap between the inferred attributes of the cancer and tumor summaries (Figs 2B and 2C) and the text of the clinical note (Fig 2G). Selection of summary items from the cancer or tumor summary lists leads to a display in the explanation panel describing the selected fact, along with information about its derivation from the given document. Like the tumor summary views, this panel illustrates the utility of the multilevel information model for moving between summary and individual observation, helping the user verify that the summarized assertion is indeed correct.

The *mention pane* (Fig 2F) provides a summary of mentions extracted from the selected document, supporting R3: Text and R4: Provenance. Each mention can be clicked to highlight the appropriate scan in the note view (Fig 2G), thus providing the user with additional tools for verifying correctness of the NLP output.

Navigation through multiple levels of abstraction is illustrated in Figure 2. The selection of tumor summary item “Ductal Breast Carcinoma in situ” (Fig 2B) led to the display of the “Invasive Ductal Carcinoma” in the explanation pane (Fig 2E) and the display of relevant mentions from Report 48 (Fig 2E). Clicking on the “positive” mention leads to text confirming the mention of invasive ductal carcinoma (Fig 2G).

## DISCUSSION

The substantial amounts of clinical text associated with histories of patients with cancer present significant challenges for retrospective research. With histories involving dozens of relevant notes, manual expert review will not be sufficient for the large-scale analyses needed to drive innovation. Although advances in cross-document coreference^26^ and other techniques currently being explored by the DeepPhe project show great promise in increasing the utility of clinical text, NLP is only a first step, providing an intermediate representation not directly consumable by end users. DeepPhe’s use of summarization and episode classification help provide order to the many facts that might be extracted from a set of patient records, but additional support is needed to turn these details into actionable understanding.

Our visualization tool is designed to tackle the four primary challenges associated with interpretation of these data: comparing patients (in the cohort view), facilitating exploration of patient histories over the time course of the available records, linking higher-level summaries to individual observations, and verifying output. Patient comparisons are necessary to enable identification of cohorts matching desired criteria. Aggregation of individual observations into higher-level clinically meaningful constructs will be necessary to easily answer key research questions such as “which patients were treated with neoadjuvant therapy?” while linkages between those aggregations and individual text mentions enable verification of results, thus building user confidence in output.

The DeepPhe visualization tool represents a first step toward these goals, providing preliminary patient and cohort views of data for patients with cancer at multiple granularities. Although limited to a subset of desired data types, the current version illustrates basic functionality needed to address key requirements (Table 2) and outstanding challenges that have been identified during the evolution of the tools. Further engagement with domain experts representing multiple classes of stakeholders will be needed to ensure alignment between user needs and system functionality,

Unlike many previous text analytics tools that focus on classification^27^ or more exploratory analysis of large text corpora,^28^ the DeepPhe tools combine NLP results with an analytics interface, thus forming a complete analytics platform. DeepPhe is perhaps most similar to HARVEST,^29^ which presents observations extracted from NLP in a timeline view. However, DeepPhe’s information model and inference rules provide support for cancer-specific higher-level abstractions not found in HARVEST. Future enhancements might include interactive features explored in related projects, including support for interactive revisions of the NLP models,^30,31^ application to federated data sets,^32^ and additional visualizations display cohort-level patterns.^12-15^

Expanding the utility of the clinical text for identifying both cohorts and individual patients may aid in the interpretive process. Improved displays for both rendering and interpreting inference rules linking higher-order abstractions to individual text mentions may be helpful for complex inferences, particularly when cross-document inference is involved. Techniques for linking observations across documents will also prove useful for identifying recurring concepts identified through cross-document coreference resolution. At the cohort level, visualization of text patterns, perhaps enhanced through a Word Tree^33^ or similar visualization, might help users interpret key phrases indicative of observations of interest.

DeepPhe visualization functionality will evolve alongside NLP capabilities. Although extraction and classification of individual mentions has led to promising results in many of the attributes currently shown in the prototype visualizations, much work remains to be done in the inference of higher-level aggregations and, subsequently, the inclusion of these representations in the visualization. Two key examples involve tumors and treatments. Linking multiple tumor references across temporal extents, and including these intervals in the timeline view, will provide valuable perspective on cancer progression and response.

Enhanced temporal aggregation will also drive extensions of the DeepPhe cohort view. Incorporation of per-document episode enhancement techniques, alongside orderings of treatments and time spans of specific tumors, will support temporally aligned cohort analysis using techniques similar to those used in Outflow,^12^ Frequence,^14^ EventFlow,^13^ and related systems.^15^ Temporal^13^ and logical^34^ search facilities are also planned, with pattern search^15,35^ a possibility for future work. Similar to previous tools focused on specific domains^36^ or care pathways and treatment plans,^37-39^ we will use episode annotations and the semantics of the DeepPhe information model to focus designs on the specific challenges of interpreting cancer data.

Inclusion of treatment information, particularly for chemotherapeutic regimens, may provide investigators with insights into treatment histories and possible impacts. Effectively displaying treatments will require inference not only of specific start and stop times of various drugs but ideally of identification or inference of multidrug protocols. Extension of DeepPhe NLP tools to identify medication regimens on the basis of the HemOnc ontology^40,41^ is a high priority.

As DeepPhe interactive tools evolve to include these new data elements, appropriate handling of uncertainty and missing information will become increasingly critical. NLP temporal modeling techniques^8,42^ might be used in combination with structured electronic health record data to eliminate some ambiguity, but many details will likely remain unspecified. Cohort and patient tools will need both appropriate display of these underspecified constraints and appropriate semantics for any related queries or filters. Temporal ambiguities also underscore the importance of tools for explicitly describing search criteria and for facilitating comparisons between cohorts as techniques that might reduce the risk of misinterpretation.

Evaluation of analytic tools such as the DeepPhe visualizations has been the subject of an active body of research. As information visualization tasks are often exploratory and ill defined, traditional metrics such as task completion time and accuracy may not be particularly informative, leading to the need for investigations into descriptions of the use of the tool in terms of analytic processes used, types of interactions, and clarity of explanations of data.^43^ Insight-based evaluations aimed at quantifying novel understanding might also be considered.^44^ Planned evaluations will follow a phased approach, combining small-scale usability visualizations with larger laboratory studies and eventual observations of the use of the tool in context.^45^ DeepPhe and the DeepPhe-Viz tool are available on GitHub.^10,24^

DeepPhe’s current architecture assumes that text files to be processed are available in a simple file structure, organized by patient. Efforts to provide DeepPhe functionality through application programming interfaces, allowing integration with other tools and data warehouse environments, are underway.

Facilitating the interpretation of complex, longitudinal patient histories is an important challenge for understanding cancer treatment and outcomes. The DeepPhe project uses a multifaceted approach, combining NLP, inference, information modeling, and interactive visualizations to provide researchers with detailed descriptions that span the gap between key phenomena of interest and specific documentary evidence. Extension of proposed prototype designs to handle richer data, particularly involving temporal spans, will set the stage for deployment with clinical researchers and subsequent evaluation studies.
